# Sexual Reproduction in *Aspergillus flavus* Sclerotia: Acquisition of Novel Alleles from Soil Populations and Uniparental Mitochondrial Inheritance

**DOI:** 10.1371/journal.pone.0146169

**Published:** 2016-01-05

**Authors:** Bruce W. Horn, Richard M. Gell, Rakhi Singh, Ronald B. Sorensen, Ignazio Carbone

**Affiliations:** 1 National Peanut Research Laboratory, Agricultural Research Service, U.S. Department of Agriculture, Dawson, Georgia, United States of America; 2 Center for Integrated Fungal Research, Program of Genetics, Department of Plant Pathology, North Carolina State University, Raleigh, North Carolina, United States of America; Georg-August-University of Göttingen Institute of Microbiology & Genetics, GERMANY

## Abstract

*Aspergillus flavus* colonizes agricultural commodities worldwide and contaminates them with carcinogenic aflatoxins. The high genetic diversity of *A*. *flavus* populations is largely due to sexual reproduction characterized by the formation of ascospore-bearing ascocarps embedded within sclerotia. *A*. *flavus* is heterothallic and laboratory crosses between strains of the opposite mating type produce progeny showing genetic recombination. Sclerotia formed in crops are dispersed onto the soil surface at harvest and are predominantly produced by single strains of one mating type. Less commonly, sclerotia may be fertilized during co-infection of crops with sexually compatible strains. In this study, laboratory and field experiments were performed to examine sexual reproduction in single-strain and fertilized sclerotia following exposure of sclerotia to natural fungal populations in soil. Female and male roles and mitochondrial inheritance in *A*. *flavus* were also examined through reciprocal crosses between sclerotia and conidia. Single-strain sclerotia produced ascospores on soil and progeny showed biparental inheritance that included novel alleles originating from fertilization by native soil strains. Sclerotia fertilized in the laboratory and applied to soil before ascocarp formation also produced ascospores with evidence of recombination in progeny, but only known parental alleles were detected. In reciprocal crosses, sclerotia and conidia from both strains functioned as female and male, respectively, indicating *A*. *flavus* is hermaphroditic, although the degree of fertility depended upon the parental sources of sclerotia and conidia. All progeny showed maternal inheritance of mitochondria from the sclerotia. Compared to *A*. *flavus* populations in crops, soil populations would provide a higher likelihood of exposure of sclerotia to sexually compatible strains and a more diverse source of genetic material for outcrossing.

## Introduction

Aflatoxins produced by *Aspergillus flavus* from section *Flavi* are among the most potent mycotoxins known. These secondary metabolites are acutely toxic to humans at high exposures and are also responsible for increased incidences of liver cancer in human populations in which contaminated food is routinely ingested [1,2]. Aflatoxin-producing fungi were originally thought to be strictly asexual in reproduction and to have lost their ability to undergo meiosis [3]. However, populations of *A*. *flavus* show high diversity in morphology, mycotoxin production and vegetative compatibility groups (VCGs) [4,5]. In addition, *A*. *flavus* populations exhibit evolutionary signatures of recombination within the aflatoxin gene cluster based on the partitioning of DNA sequence variation into distinct linkage disequilibrium blocks [6,7]. The discovery of sexual reproduction in *A*. *flavus* [8] in laboratory crosses as well as the demonstration of independent assortment of chromosomes and crossing over [9,10] suggest that sexuality is largely responsible for the genetic variation observed in natural populations. For example, many *A*. *flavus* strains in populations do not produce aflatoxins due to specific deletions in the aflatoxin gene cluster [11]. The locations of these deletions were shown to correspond to cross over points during meiosis in laboratory crosses [9]. Therefore, sexual reproduction and genetic recombination in nature may be responsible for the genetic variation among nonaflatoxigenic *A*. *flavus* strains.

*A*. *flavus* is heterothallic, with individuals containing one of two mating-type alleles, *MAT1-1* and *MAT1-2* [7,12]. Sexual reproduction in crosses between opposite mating types is characterized by the formation of indehiscent ascospore-bearing ascocarps within the matrix of sclerotia [8]. In many fungi, sexual reproduction is also regulated by a sex-based (female/male) mating system independent of mating type [13–15], but such as system has not been reported in *A*. *flavus*. Two morphotypes of *A*. *flavus* based on sclerotial size have been described: the L (large) strain with sclerotia > 400 μm diam and the S (small) strain with sclerotia < 400 μm [16]. Sclerotia are readily produced by single strains in culture [5] and in wound-inoculated crops [17,18], and their formation is not dependent on mating; hence, they are primarily considered to be survival structures for withstanding adverse environmental conditions [19].

Sclerotia of *A*. *flavus* are naturally produced in crops [20,21] and are dispersed onto the soil surface during harvest [20]. The majority of these sclerotia likely originate from single strains of one mating type. To examine the capacity of naturally formed *A*. *flavus* sclerotia to produce the sexual stage, Horn et al. [21] collected sclerotia from corn exposed to different levels of drought stress over a 3-year period. There was no evidence of ascocarp and ascospore formation in sclerotia at corn harvest, but incubation of sclerotia on the surface of soil in the laboratory resulted in ascospore formation in a very small percentage of sclerotia. Horn et al. [21] postulated that fertilization occurred in the crop and that the development of sexual structures occurred after dispersal of sclerotia onto the soil surface at harvest. The low incidence of sexual reproduction in *A*. *flavus* sclerotia was attributed to the low probability of co-infection of corn with sexually compatible strains. Therefore, although fertilized sclerotia may be dispersed onto soil, the majority of sclerotia will be unfertilized and consist of single strains.

In this study, laboratory and field experiments were performed to examine the capacity of single-strain sclerotia of one mating type and fertilized sclerotia that had not yet formed ascocarps to produce ascospores on soil containing natural fungal populations. Progeny were examined for recombination and the presence of novel parental alleles. Reciprocal crosses between sclerotia and conidia were also performed to investigate female and male roles and mitochondrial inheritance in *A*. *flavus* sexual reproduction. This research shows that both single-strain and fertilized sclerotia can undergo sexual development on soil, but progeny from single-strain sclerotia contain novel alleles from fertilization by soil strains, whereas progeny from fertilized sclerotia contain only known parental alleles. Furthermore, reciprocal crosses between sclerotia and conidia show that *A*. *flavus* is hermaphroditic with respect to female and male roles in sexual reproduction and that inheritance of mitochondria is uniparental.

## Materials and Methods

### Fungal strains and sclerotium production

*A*. *flavus* L strains used to produce sclerotia (Table 1) were chosen based on mating type and high fertility in laboratory crosses [8,9,12]. Nonaflatoxigenic biocontrol strains NRRL 21882 from Afla-Guard and AF36 (= NRRL 18543), both used commercially for reducing aflatoxins in crops [22], were assigned to VCGs according to Horn and Dorner [23] and Ehrlich et al. [24], respectively. The remaining strains were obtained from soil and peanut seeds from a field (private land with permission) in Terrell Co., Georgia (31°41’39”N 84°25’00”W), and were previously characterized by VCG [4]; all strains produce aflatoxin B_1_ and cyclopiazonic acid [5].

**Table 1:** Sexual reproduction in *A*. *flavus* sclerotia from single strains of one mating type when incubated on sterile soil and on soil containing natural fungal populations.

Sclerotium-producing strain^a^			Sterile soil		Nonsterile soil		
*MAT1-1*	*MAT1-2*	VCG	No. sclerotia examined	% fertile sclerotia^b^	No. sclerotia examined	% fertile sclerotia^b^	No. progeny^c^
29507		33	300	0	300	58.7 ± 10.7^d^	36
29473		17	300	0	300	56.0 ± 2.6^d^	36
29537		63	300	0	718	0.1 ± 0.2^e^	12
	29536	62	300	0	312	80.2 ± 8.0	36
	21882	24	300	0	688	0.4 ± 0.8^e^	36
	29487	25	300	0	673	0^e^	
	AF36	YV36	300	0	674	0^e^	

^a^Strain numbers (except AF36) from Agricultural Research Service Culture Collection (NRRL), Peoria, Illinois. Nonaflatoxigenic biocontrol strains include NRRL 21882 (from Afla-Guard) and AF36 (= NRRL 18543); all other strains produce aflatoxin B_1_ and cyclopiazonic acid [5]. Mating-type designations from Ramirez-Prado et al. [12] and vegetative compatibility groups (VCGs) based on Horn and Greene [4] except for YV36 [24].

^b^Sclerotia with ascospores considered fertile; means ± SD based on three soil cups.

^c^Progeny obtained from three ascocarps in separate sclerotia (n = 12 progeny per ascocarp) except for NRRL 29537, which produced a single ascocarp.

^d,e^Pairwise chi-square test of independence failed to differentiate the fertility of these isolates (P > 0.05) (S1 Text).

For producing sclerotia, slants containing mixed cereal agar (MCA) [25] were inoculated with conidia from either single strains or pairs of strains in crosses according to Horn et al. [26]. Cultures were incubated in darkness for 14 d at 30°C, at which time fertilized sclerotia from crosses had not yet formed ascocarps. Since the fertilization process has not been observed in *A*. *flavus*, sclerotia were considered to be fertilized based on the subsequent development of ascocarps with ascospores under laboratory conditions. In all crosses, a certain percentage of sclerotia did not form the sexual stage. Sclerotia from single strains and crosses were harvested and then air dried and stored in a desiccator over saturated NaCl solution at 25°C (75% relative humidity) [26]. Following a subsequent incubation period in the laboratory or field, sclerotia were surface sterilized, dissected with a microscalpel, and examined for ascocarps with the stereomicroscope. To obtain progeny, ascospores were removed from individual ascocarps with a microneedle and dilution plated on malt extract agar containing 30 mg/L streptomycin and 1.5 mg/L chlortetracycline [26]. Germlings were observed with the light microscope (200×) after 20–24 h incubation at 30°C and transferred to Czapek agar (CZ).

### Laboratory incubation of single-strain sclerotia on soil

The capacity of single-strain sclerotia of one mating type to produce ascospores through incubation on soil containing natural soil populations was examined under laboratory conditions. Soil was collected 17 March 2011 from a cornfield 1.1 km southeast of Shellman, Randolph Co., Georgia (Field A) (31°44’47”N 84°36’22”W). Soil sampling and field trials were conducted on property owned or leased by ARS-USDA. Soil was air dried to 1.6 ± 0.01% moisture (± SD, n = 3; dry weight basis) and sieved through No. 12 and 20 Standard Testing Sieves in tandem. Potential mating population densities of *A*. *flavus* in soil were determined by suspending each of three subsamples (33 g) in 100 mL 0.2% water agar and dilution plating onto five plates of modified dichloran-rose Bengal medium (mDRB) [27]. Plates were incubated for 3 d at 37°C and section *Flavi* species were identified according to Horn and Dorner [27]. Soil population densities were calculated on a dry-weight soil basis. In addition, 37 randomly selected *A*. *flavus* L strain colonies from soil dilution plates were single spored for mating-type determination.

Sieved soil was mixed with sterile distilled water (14 mL per 100 g) and allowed to equilibrate overnight in a sealed container before adding to 30-cm^3^ plastic medicine cups [21]. *A*. *flavus* sclerotia from each of seven single strains (Table 1) were added to the soil surface of three medicine cups (approximately 300 per cup). Sclerotia were similarly added to cups containing autoclaved soil to which sterile water (14 mL per 100 g) had been added. Cups were incubated separately in darkness for 6 mo on shallow platforms over distilled water in wide-mouth sealed quart jars (30°C; 100% relative humidity).

### Field incubation of single-strain and fertilized sclerotia

The capacity to produce ascospores by single-strain sclerotia of one mating type and fertilized sclerotia that had not yet formed ascocarps was examined in three fields. Single-strain and fertilized sclerotia were applied to the soil surface of three non-irrigated cultivated fields in Georgia: Field A, described above near Shellman; Field B, 4.8 km northwest of Dawson, Terrell Co. (31°47’06”N 84°29’16”W); and Field C, 4.8 km southeast of Dawson, Terrell Co. (31°43’59”N 84°23’37”W). Soil from Field B was Faceville fine sandy loam (fine, kaolinitic, thermic Typic Kandiudults) and soils from Fields A and C were Greenville fine sandy loam (fine, kaolinitic, thermic Rhodic Kandiudults). Analyses for soil texture, organic matter and pH were performed by Waters Agricultural Laboratories, Camilla, GA. Rainfall and air temperatures were recorded from onsite electronic weather stations (Campbell Scientific, Logan, UT). Daily minimum, maximum and mean temperature values for each month (n = 28–31 except n = 15 for April) were statistically compared among fields with ANOVAs followed by Student-Newman-Keuls (SNK) test for comparison of means.

A 16 × 6 m plot was delimited and fenced within each field. Within each plot, 13 circular white PVC rings (15.2 cm diam and deep) were spaced approximately 2 m apart and inserted 7–8 cm into the soil. To determine potential mating population densities of *A*. *flavus* in the plot soils, five soil samples (33 g each) from the top 3 cm were randomly collected outside of the rings from each plot on 16 Apr 2013 (immediately before applying sclerotia) and were dilution plated on mDRB as described above. Population densities of section *Flavi* species in the three fields were statistically compared with ANOVAs followed by SNK test for comparison of means. Twelve randomly selected *A*. *flavus* L strain colonies from each field were single spored for mating-type determination.

Single-strain sclerotia and fertilized sclerotia from crosses were harvested from MCA slants (14 d; 30°C) for applying to the fields. Previous research [21] demonstrated that *A*. *flavus* sclerotia from crosses did not contain ascocarps after 14 d. However, a certain percentage of sclerotia contained ascocarps with free ascospores after 4 mo when sclerotia were incubated in MCA slants and on soil in the laboratory. To ensure that sclerotia from crosses in this study were also capable of forming the sexual stage when applied to the fields, sclerotia in MCA slants were incubated an additional 4 mo at 30°C in sealed plastic bags [26] and sclerotia harvested from MCA slants (14 d; 30°C) were incubated on the surface of nonsterile soil in cups within fruit jars for 4 mo at 30°C as described above. Approximately 1000–1500 sclerotia from each of seven single strains and six sexually compatible crosses (Table 2) were sprinkled onto the soil surface within randomly selected rings. Distilled water (100 mL) was applied to each ring with a watering can immediately following application of sclerotia; thereafter, sclerotia were exposed wholly to rainfall. Pesticides were not applied to the plots, and weeds were allowed to grow freely to form a canopy over the rings.

**Table 2:** Sexual reproduction in single-strain and fertilized sclerotia of *A*. *flavus* under field conditions.

Strain(s)^a^		Field A			Field B			Field C		
		No. sclerotia examined^b^	No. fertile sclerotia^c^	No. progeny^d^	No. sclerotia examined^b^	No. fertile sclerotia^c^	No. progeny^d^	No. sclerotia examined^b^	No. fertile sclerotia^c^	No. progeny^d^
*MAT1-1*	*MAT1-2*									
Single-strain sclerotia										
29507		624 (74.3)	0		673 (88.9)	8 (1.2)	108	230 (96.2)	0	
29473		582 (69.3)	0		342 (92.0)	2 (0.6)	59	225 (95.2)	0	
29537		631 (68.0)	0		540 (56.0)	1 (0.2)	1	444 (42.7)	0	
	29536	407 (90.5)	0		281 (80.3)	0		423 (64.2)	0	
	21882	1273 (38.7)	0		917 (77.6)	0		671 (81.3)	0	
	29487	761 (84.4)	0		219 (73.8)	0		509 (98.7)	0	
	AF36	1117 (69.3)	0		517 (64.0)	0		414 (86.0)	0	
Fertilized sclerotia from crosses										
29473 × 29487		762 (88.0)	3 (0.4)	36	988 (84.0)	1 (0.1)	12	356 (94.8)	2 (0.6)	24
29537 × 29536		108 (45.2)	0		254 (38.2)	0		173 (59.0)	0	
29507 × AF36		660 (33.3)	8 (1.2)	36	475 (36.0)	8 (1.7)	36	382 (18.7)	11 (2.9)	36
29473 × AF36		875 (65.3)	79 (9.0)	36	523 (72.0)	17 (3.3)	36	198 (83.3)	8 (4.0)	36
29507 × 21882		442 (96.0)	49 (11.1)	36	581 (77.3)	36 (6.2)	36	317 (84.0)	72 (22.7)	36
29473 × 21882		514 (62.7)	0		343 (64.0)	2 (0.6)	24	288 (60.9)	3 (1.0)	36

^a^Strain numbers (except AF36) from Agricultural Research Service Culture Collection (NRRL), Peoria, Illinois; mating-type designations from Ramirez-Prado et al. [12]; vegetative compatibility group designations are shown in Table 1. Biocontrol strains include NRRL 21882 (from Afla-Guard) and AF36 (= NRRL 18543).

^b^Percentage viability of sclerotia (n = 49–75) in parentheses.

^c^Sclerotia with ascospore-bearing ascocarps considered fertile; percentage of sclerotia that were fertile in parentheses.

^d^In single-strain sclerotia from Field B, variable numbers of progeny were obtained from ascocarps in all available fertile sclerotia; in fertilized sclerotia from crosses in Fields A-C, progeny were obtained from 1–3 ascocarps in separate sclerotia (n = 12 progeny per ascocarp).

The top 2 cm of soil was removed from the entire area within each ring after incubation of sclerotia for one year (April 2014). Soil was added to a 1-L graduated cylinder and brought to a final volume of 1 L with 2.5 M sucrose [28]. After vigorously shaking the cylinder, sclerotia and other organic matter were allowed to float to the top of the sucrose solution (3 h). The floating fraction was then transferred to a 100-mesh filter, rinsed with distilled water, and retransferred to 9-cm Whatman #1 filter paper from which sclerotia were removed using a stereomicroscope. Sclerotia were surface sterilized for 2 min with 0.25% sodium hypochlorite and rinsed before dissection. Dissected sclerotia without ascocarps from each ring (n = 49–75) were plated on CZ with antibiotics to test for viability.

### Reciprocal crosses between single-strain sclerotia and conidia

Reciprocal crosses between sclerotia and conidia were performed in the laboratory to examine female and male roles in sexual reproduction and to determine the pattern of mitochondrial inheritance. *A*. *flavus* sclerotia from single strains of one mating type were added to medicine cups containing autoclaved sieved soil to which conidia of the opposite or same mating type had been added. Conidia were obtained by inoculating slants containing CZ with 400g/L sucrose and incubating in darkness for 14 d at 30°C. Sterile glass beads (2.5 g; 90–150 μm diam) were then added to each slant and shaken to coat the beads with conidia [29]. To effectively mix the conidia in soil, coated beads (0.09 g) for each strain were added to a jar containing 600 g of dry autoclaved soil and thoroughly shaken. Inoculated soil (3.3 g) was added to 10 mL water agar and dilution plated on mDRB plates (3 d; 37°C) to determine fungal density; soil in jars was then adjusted with sterile soil or conidia-coated beads to attain approximately 2000 CFU/g. Sterile water was added to the inoculated soil (14 mL per 100 g) and sclerotia were incubated in soil cups within sealed fruit jars for 6 mo at 30°C as described above. Three pairs of reciprocal sclerotia-conidia combinations (Table 3) were set up using crosses that produced sclerotia with high fertility when incubated 4 mo in MCA slants and soil cups (Table 4). For each reciprocal pair, sclerotia of the *MAT1-1* strain were incubated on soil containing conidia of the *MAT1-2* strain, and sclerotia of the *MAT1-2* strain were incubated on soil containing conidia of the *MAT1-1* strain. In addition, sclerotia of the two strains from each reciprocal pair were incubated with conidia of a strain of the same mating type (*MAT1-1* or *MAT1-2*).

**Table 3:** Reciprocal crosses in *A*. *flavus* in which single-strain sclerotia were incubated on sterilized soil inoculated with conidia.

Sclerotia on soil^a^			Conidia in sterilized soil^a^				
*MAT1-1*	*MAT1-2*		*MAT1-1*	*MAT1-2*	No. sclerotia examined	% fertile sclerotia^b^	No. progeny^c^
29537		×		29536	480	1.1 ± 0.9^d^	36
	29536	×	29537		300	98.7 ± 0.6^d^	36
29537		×	29507		300	0	
	29536	×		AF36	300	0	
29507		×		21882	300	96.7 ± 2.3^e^	35
	21882	×	29507		300	1.0 ± 0.0^e^	36
29507		×	29473		300	0	
	21882	×		29536	300	0	
29473		×		AF36	300	85.7 ± 0.6^f^	36
	AF36	×	29473		300	27.7 ± 12.5^f^	36
29473		×	29537		300	0	
	AF36	×		21882	300	0	

^a^Strain numbers (except AF36) from Agricultural Research Service Culture Collection (NRRL), Peoria, Illinois; mating-type designations from Ramirez-Prado et al. [12]; vegetative compatibility group designations are shown in Table 1. Biocontrol strains include NRRL 21882 (from Afla-Guard) and AF36 (= NRRL 18543).

^b^Sclerotia with ascospores considered fertile; means ± SD based on three soil cups.

^c^Progeny obtained from three ascocarps in separate sclerotia (n = 11–12 progeny per ascocarp).

^d,e,f^Reciprocal crosses within each pair significantly different (P < 0.0001) according to chi-square test of independence (S1 Text).

**Table 4:** Sexual reproduction in *A*. *flavus* sclerotia obtained from crosses and incubated under laboratory conditions.

Cross^a^		Culture slants (14 d)^b^		Culture slants (4 mo)		Nonsterile soil cups (4 mo)	
*MAT1-1*	*MAT1-2*	No. sclerotia examined	% fertile sclerotia	No. sclerotia examined	% fertile sclerotia^c^	No. sclerotia examined	% fertile sclerotia^c^
29473 × 29487		1050	0	600	14.7 ± 5.6	240	42.8 ± 8.6
29537 × 29536		851	0	600	50.5 ± 8.4^d^	240	61.1 ± 23.5
29507 × AF36		1004	0	499	91.5 ± 3.8	240	78.0 ± 23.5^e^
29473 × AF36		750	0	576	76.0 ± 5.5	240	71.0 ± 14.6
29507 × 21882		1050	0	600	54.5 ± 12.8^d^	210	78.3 ± 3.4^e^
29473 × 21882		1002	0	600	24.8 ± 21.0	240	24.9 ± 10.5

^a^Strain numbers (except AF36) from the Agricultural Research Service Culture Collection (NRRL), Peoria, Illinois; AF36 (= NRRL 18543) and NRRL 21882 are nonaflatoxigenic biocontrol strains.

^b^Sclerotia harvested for adding to nonsterile soil cups (shown in this table) and to Fields A-C (Table 2).

^c^Sclerotia with ascospores considered fertile; means ± SD based on six MCA slants or soil cups.

^d,e^Pairwise chi-square test of independence failed to differentiate the fertility of these isolates; all other values were significantly different (*P* < 0.01) (S1 Text).

### Mating-type determination and genotype analyses

DNA was extracted from *A*. *flavus* soil and progeny strains as previously described [30]; previously generated sequence data [9] were used for parental strains. Mating types for strains were determined according to Ramirez-Prado et al. [12]. Deletion types for the *aflF-aflU* region were determined according to Chang et al. [11]. PCR amplification for the *AF17* and *AF48* microsatellite loci was performed based on Grubisha and Cotty [31] and for *aflC* based on Moore et al. [6]. Oligonucleotides for *AF-MIT-1* (F: TGAAGCAACTGGATTATTCGCA, R: AAACCACATTCAAAAGCGCT), *AF-MIT-3* (F: AGCAGAGGGTTCTGCGTTT, R: GCAGATCAACCTGCTAATAATATTCC) and *AF-MIT-4* (F: GCTAAAGTTATAGGAGGTGAAGT, R: GCAACCTTTAGCTTCAATAAACCC) were designed for amplification of polymorphic loci in the mitochondrial genomes of parental and progeny strains. Nuclear *AF17*, *AF48* and *aflC* and mitochondrial amplicons were sequenced by the North Carolina State University Genomic Sciences Laboratory and aligned using SEQUENCHER version 4.7 (Gene Codes Corporation, Ann Arbor, MI). Haplotypes were designated based on single-nucleotide polymorphisms, insertion/deletion events and trinucleotide repeat lengths using the SNAP Map and Combine programs [32] implemented in Mobyle SNAP Workbench [33,34].

Three ascocarps from different sclerotia, when possible, were randomly chosen from single-strain sclerotia that became fertilized following incubation on soil in the laboratory (NRRL 29507, NRRL 29473, NRRL 29536) or in the field (NRRL 29507, NRRL 29473). Three progeny strains from each ascocarp were analyzed at the *MAT* locus on chromosome 6, *AF17* locus on chromosome 2, *AF48* locus on chromosome 7, and *aflF-aflU* intergenic region on chromosome 3. For fertilized sclerotia obtained from crosses between known parental strains (NRRL 29507 × AF36, NRRL 29473 × AF36, NRRL 29507 × NRRL 21882) and incubated under field conditions, two ascocarps from different sclerotia were randomly chosen per field. Three progeny strains from each ascocarp were analyzed at the *MAT*, *AF17*, *AF48* and *aflC* (chromosome 3) loci. For the three pairs of reciprocal crosses between sclerotia and conidia from known parents, three ascocarps per cross from different sclerotia were chosen. Two progeny strains per ascocarp were analyzed at the *MAT* locus and a mitochondrial marker (*AF-MIT-1* for NRRL 29537 and NRRL 29536; *AF-MIT-3* for NRRL 29507 and NRRL 21882; and *AF-MIT-4* for NRRL 29473 and AF36).

## Results

### Laboratory incubation of single-strain sclerotia on soil

Soil used for laboratory incubation of sclerotia from single strains of one mating type contained a sizable potential mating population of *A*. *flavus* L strain. Section *Flavi* species included: *A*. *flavus* L strain (777 ± 216 CFU/g; ± SD, n = 3), *A*. *flavus* S strain (11 ± 4), *A*. *parasiticus* (149 ± 27), *A*. *caelatus* (508 ± 117) and *A*. *tamarii* (14 ± 6). Of the 37 *A*. *flavus* L strains randomly sampled from the soil dilution plates, 19 (51%) were *MAT1-1* and 18 (49%) were *MAT1-2*.

Sclerotia from three *MAT1-1* and four *MAT1-2* single strains of *A*. *flavus* were incubated for 6 mo on the surface of sterile soil and soil containing natural fungal populations (Table 1). Sclerotia incubated on sterile soil showed no evidence of ascocarp formation, whereas sclerotia from five of the strains (NRRL 29507, NRRL 29473, NRRL 29537, NRRL 29536 and NRRL 21882) incubated on soil with natural fungal populations showed ascospore formation in 0.1–80.2% of sclerotia; two of the strains (NRRL 29487 and AF36) did not produce ascocarps (Table 1, S1 Text). Of the progeny examined from single-strain sclerotia (NRRL 29507, NRRL 29473, NRRL 29536), only 2 of 27 (IC5210 and IC5958) showed multilocus sequence types (MLSTs) that matched the known sclerotial parent (Table 5). The remaining progeny showed biparental inheritance with independent assortment of chromosomes and contained novel alleles from wild strains in soil. Sequence polymorphisms in *AF17* and *AF48* identified the novel alleles contributed by wild parents. Progeny from each of the nine ascocarps examined showed inheritance from a single wild strain; ascocarps 1 and 3 from NRRL 29536 had MLSTs consistent with the same wild parental strain (Table 5). Both mating-type alleles (*MAT1-1/MAT1-2*) were detected in three of the progeny. Progeny strains from the incubation of NRRL 21882 sclerotia could not be conclusively genotyped due to the presence of multiple alleles inherited from the known parental strain and wild strains. All sequence data for *AF17* and *AF48* were submitted to GenBank under Accession numbers KR922515- KR922572.

**Table 5:** Genotype data for *A*. *flavus* progeny from single-strain sclerotia fertilized by strains from natural soil populations when incubated under laboratory conditions.

Strain^a^	Loci examined			
	Chr. 6 *MAT*	Chr. 2 *AF17*	Chr. 7 *AF48*	Chr. 3 *aflF/aflU*
**Parents**				
NRRL 29507	1	H1	H1	III
Wild strain A^b^	2	H2	H2	I
Wild strain B^b^	2	H3	H3	II
Wild strain C^b^	2	H4	H4	II
**Progeny**				
Ascocarp 1				
IC5956	2	H2	H1	III
IC5957	1	H1	H2	I
IC5958	1	H1	H1	III
Ascocarp 2				
IC5968	1	H3	H3	-^c^
IC5969	1	H3	H3	-^c^
IC5970	1	H1	H3	II
Ascocarp 3				
IC5980	2	H4	H4	II
IC5981	2	H1	H1	III
IC5982	2	H1	H1	III
**Parents**				
NRRL 29473	1	H1	H1	III
Wild strain A^b^	2	X^d^	H2	I
Wild strain B^b^	2	X^d^	H3	I
Wild strain C^b^	2	H4	X^d^	I
**Progeny**				
Ascocarp 1				
IC5151	2	H1	H2	I
IC5152	2	H1	H1	I
IC5154	1	H1	H1	-^c^
Ascocarp 2				
IC5164	1	H1	H3	III
IC5166	1&2	H1	H1	III/I^e^
IC5167	1	H1	H3	-^c^
Ascocarp 3				
IC5186	2	H1	H1	I
IC5188	1	H4	H1	I
IC5189	1	H1	H1	I
**Parents**				
NRRL 29536	2	H1	H1	III
Wild strain A^b^	1	H2	H2	I
Wild strain B^b^	1	H3	X^d^	II
**Progeny**				
Ascocarp 1^f^				
IC5197	1&2	H2	H2	III
IC5198	1	H2	H1	I
IC5201	1	H2	H2	III
Ascocarp 2				
IC5209	2	H3	H1	III
IC5210	2	H1	H1	III
IC5211	1&2	H1	H1	II
Ascocarp 3^f^				
IC5221	1	H2	H2	I
IC5222	1	H2	H2	I
IC5223	2	H2	H1	III

^a^NRRL strain numbers from the Agricultural Research Service Culture Collection, Peoria, Illinois, and IC strain numbers from the Ignazio Carbone Culture Collection, North Carolina State University, Raleigh, North Carolina.

^b^MLSTs of wild strains determined by identification of alleles in progeny differing from those of the known sclerotial strain.

^c^PCR did not amplify.

^d^Allele not seen in tested progeny.

^e^PCR yielded two amplicons corresponding to deletion types III and I.

^f^Ascocarps 1 and 3 fertilized by wild strain with the same MLST.

### Field incubation of single-strain and fertilized sclerotia

Soil within each field plot immediately prior to application of single-strain and fertilized sclerotia contained native populations of *A*. *flavus* L strain potentially capable of fertilizing the sclerotia. Populations from section *Flavi* included *A*. *flavus* L and S strains, *A*. *parasiticus*, *A*. *tamarii*, *A*. *caelatus* and *A*. *alliaceus* (Table 6). *A*. *flavus* L strain densities in Fields A and C (398 and 391 CFU/g, respectively) were not significantly different (P = 0.96), whereas the density in Field B (8135 CFU/g) was significantly greater than those of Fields A and C (P < 0.0001). Mating types of randomly selected isolates (n = 12) from soil were: 7 (58%) *MAT1-1* and 5 (42%) *MAT1-2* for Field A; 6 (50%) *MAT1-1* and 6 (50%) *MAT1-2* for Field B; and 2 (17%) *MAT1-1*, 9 (75%) *MAT1-2* and 1 (8%) *MAT1-1/MAT1-2* for Field C. Sclerotia within rings in the plots were initially unshaded when applied in April 2013 but were covered by weeds by June.

**Table 6:** Soil populations of *Aspergillus* section *Flavi* species (CFU/g) in fields prior to application of *A*. *flavus* sclerotia.^a^

Species	Field A	Field B	Field C
*A*. *flavus* L strain	398 ± 230	8135 ± 1884 ^b^	391 ± 173
*A*. *flavus* S strain	20 ± 30	136 ± 111 ^b^	40 ± 21
*A*. *parasiticus*	83 ± 48	179 ± 68	255 ± 375
*A*. *tamarii*	0	4 ± 8	260 ± 183 ^b^
*A*. *caelatus*	4 ± 5 ^b^	18 ± 13 ^b^	654 ± 164 ^b^
*A*. *alliaceus*	1025 ± 788 ^b^	7 ± 10	13 ± 15

^a^Means ± SD based on five soil samples.

^b^Density of the species or strain significantly different (*P* < 0.05) from the other fields based on ANOVA and SNK comparison of means.

Soil analyses for Field B showed 81.2% sand, 10.4% clay, 8.4% silt and 0.8% organic matter; Fields A and C were similar for percentage sand (67.2 and 69.2, respectively), clay (20.0, 20.4), silt (12.8, 10.4) and organic matter (1.2, 1.0). The pH of soil from the three fields was 6.6–7.2. During the course of the experiment, monthly maximum, minimum and mean air temperatures (S1 Table) showed no significant differences (P > 0.05) among the three fields, with the exception of minimum temperature in July 2013 in which Field A was significantly lower (P < 0.0001) than Fields B and C. Sclerotia in the three fields were exposed to air temperatures below freezing for 3–5 d in Nov, 4 d in Dec, 18–19 d in Jan, 5–6 d in Feb and 1–2 d in March. Rainfall totals for one year following application of sclerotia to Fields A, B and C were 151.4, 146.0 and 167.0 cm, respectively (S1 Table). Monthly variations in rainfall among fields were primarily due to thunderstorms in which rainfall can be intense and localized.

Single-strain sclerotia of one mating type when dissected after one year in the field showed a low frequency of ascospore formation (≤ 1.2%) in Field B but not Fields A and C (Table 2, S1 Text). Ascospores were present in sclerotia of three *MAT1-1* strains (NRRL 29507, NRRL 29473, NRRL 29537). Viability of sclerotia that did not form ascocarps in the three fields ranged from 38.7 to 98.7%; nonviable sclerotia were often colonized by *Fusarium* species. Sclerotia of NRRL 29507 and NRRL 29473 produced relatively few progeny strains that matched the MLST of the known parental strain; three of the progeny contained both mating-type alleles (*MAT1-1/MAT1-2*) (Table 7). In the majority of progeny, MLSTs indicated biparental inheritance and independent assortment of chromosomes. Sequence polymorphisms in *AF17* and *AF48* distinguished novel alleles contributed by wild parents from soil. Each ascocarp showed inheritance from a single wild strain; progeny from ascocarps 2A and 2B within the same sclerotium of NRRL 29473 appear to have originated from fertilization by the same wild strain (Table 7).

**Table 7:** Genotype data for *A*. *flavus* progeny from single-strain sclerotia fertilized by strains from natural soil populations in the field.

Strain^a^	Loci examined			
	Chr. 6 *MAT*	Chr. 2 *AF17*	Chr. 7 *AF48*	Chr. 3 *aflF/aflU*
**Parents**				
NRRL 29507	1	H1	H1	III
Wild strain A^b^	2	H2	H2	II
Wild strain B^b^	2	H3	H3	II
Wild strain C^b^	2	H4	H4	II
**Progeny**				
Ascocarp 1				
IC9156	2	H2	H2	III
IC9157	1	H1	H2	II
IC9158	1	H2	H1	III
Ascocarp 2				
IC9168	2	H3	H3	III
IC9169	1	H1	H1	III
IC9170	1	H1	H1	II
Ascocarp 3				
IC9180	1	H1	H4	III
IC9181	2	H1	H1	II
IC9182	2	H4	H4	III
**Parents**				
NRRL 29473	1	H1	H1	III
Wild strain A^b^	2	H2	H2	II
Wild strain B^b^	2	X^c^	X^c^	II
**Progeny**				
Ascocarp 1				
IC10107	2	H2	H2	III
IC10108	1	H1	H1	II
IC10109	2	H2	H1	III
Ascocarp 2A^d^				
IC10119	1&2	H1	H1	II
IC10120	1&2	H1	H1	III
IC10123	2	H1	H1	II
Ascocarp 2B^d^				
IC10148	1&2	H1	H1	III
IC10149	1	H1	H1	III
IC10152	1	H1	H1	II

^a^NRRL strain numbers from the Agricultural Research Service Culture Collection, Peoria, Illinois, and IC strain numbers from the Ignazio Carbone Culture Collection, North Carolina State University, Raleigh, North Carolina.

^b^MLSTs of wild strains determined by identification of alleles in progeny differing from those of the known sclerotial strain.

^c^Allele not seen in tested progeny.

^d^Ascocarps 2A and 2B from the same sclerotium; fertilized by wild strain with the same MLST.

Fertilized sclerotia from all six crosses did not contain ascocarps when harvested from MCA slants (14 d; 30 C) prior to application to the three fields (Table 4). In all crosses, a certain percentage of sclerotia were capable of sexual reproduction at the time of field application. Sclerotia formed ascospores after extended incubation (4 mo) in MCA slants (14.7–91.5% fertility) and on the surface of nonsterile soil under laboratory conditions (24.9–78.3%) (Table 4). Fertilized sclerotia from laboratory crosses, with the exceptions of NRRL 29537 × 29536 (Fields A-C) and NRRL 29473 × 21882 (Field A), showed low frequencies of ascospore formation in the three fields (Table 2). In all fields, sclerotia from NRRL 29507 × 21882 were most fertile (6.2–22.7%) followed by NRRL 29473 × AF36 (3.3–9.0%) and NRRL 29507 × AF36 (1.2–2.9%). When incubated in MCA slants and soil cups, sclerotia from these three crosses also showed significantly higher fertilities than sclerotia from the other crosses when compared with the chi-square test of independence (P < 0.01), except for NRRL 29507 × 21882 compared to NRRL 29537 × NRRL 29536 when incubated in MCA slants (P = 0.17) (Table 4, S1 Text). Viability of sclerotia that did not form ascocarps in the three fields ranged from 18.7 to 96.0% (Table 2). Fertile ascocarps from sclerotia of NRRL 29507 × AF36, NRRL 29473 × AF36 and NRRL 29507 × NRRL 21882 in all three fields produced progeny showing independent assortment of chromosomes (Table 8). Progeny strains from the laboratory-fertilized sclerotia contained only known parental alleles; no novel alleles from wild strains were detected (Table 8). Sequence data for *aflC*, *AF17* and *AF48* were submitted to GenBank under Accession numbers KR922585- KR922776.

**Table 8:** Genotype data for *A*. *flavus* progeny obtained from fertilized sclerotia that were applied to fields before ascocarp formation.

Strain^a^	Loci examined			
	Chr. 6 *MAT*	Chr. 2 *AF17*	Chr. 7 *AF48*	Chr. 3 *aflC*
**Parents**				
NRRL 29507	1	H1	H1	H1
AF36	2	H2	H2^b^	H2
**Progeny**				
*Field A*				
Ascocarp 1				
IC9312	1	H2	H2^b^	H1
IC9313	1	H1	H1	-^c^
IC9316	1	H2	H1	-^c^
Ascocarp 2				
IC9324	2	H2	H1	H2
IC9326	2	H2	H2^b^	H2
IC9327	2	H1	H1	H1
*Field B*				
Ascocarp 1				
IC9452	1	H1	H2^b^	H2
IC9453	2	H1	H1	H2
IC9455	1	H2	H2^b^	H2
Ascocarp 2				
IC9464	2	H2	H2^b^	H2
IC9467	2	H1	H1	H1
IC9468	1	H1	H1	H2
*Field C*				
Ascocarp 1				
IC10222	2	H1	H2^b^	H2
IC10223	1	H1	H2^b^	H1
IC10226	1	H1	H2^b^	H2
Ascocarp 2				
IC10235	1	H1	H2^b^	H1
IC10236	1	H1	H2^b^	H2
IC10237	2	H1	H2^b^	H1
**Parents**				
NRRL 29473	1	H1	H1	H1
AF36	2	H2	H2^b^	H2
**Progeny**				
*Field A*				
Ascocarp 1				
IC9348	2	H1	H2^b^	H1
IC9349	2	H1	H2^b^	H2
IC9350	1	H1	H2^b^	H1
Ascocarp 2				
IC9360	2	H1	H2^b^	H2
IC9361	1	H1	H2^b^	H1
IC9362	1	H1	H1	H1
*Field B*				
Ascocarp 1				
IC9488	1	H1	H2^b^	H1
IC9489	1	H1	H1	H1
IC9490	2	H1	H2^b^	H2
Ascocarp 2				
IC9512	2	H1	H1	H1
IC9513	2	H1	H2^b^	H2
IC9514	1	H1	H2^b^	H2
*Field C*				
Ascocarp 1				
IC10258	1	H1	H1	H2
IC10259	2	H1	H2^b^	H2
IC10260	2	H1	H1	H2
Ascocarp 2				
IC10270	2	H1	H1	H2
IC10271	2	H1	H2^b^	H2
IC10279	2	H1	H2^b^	H2
**Parents**				
NRRL 29507	1	H1	H1	H1
NRRL 21882	2	H2	H2	-^d^
**Progeny**				
*Field A*				
Ascocarp 1				
IC9384	1	H2	H2	H1
IC9385	2	H2	H1	H1
IC9386	1	H2	H2	- ^d^
Ascocarp 2				
IC9396	2	H2	H2	- ^d^
IC9397	2	H2	H1	H1
IC9398	2	H2	H1	H1
*Field B*				
Ascocarp 1				
IC9524	1	H2	H1	- ^d^
IC9525	2	H2	H2	H1
IC9526	2	H2	H1	H1
Ascocarp 2				
IC9536	1	H1	H2	H1
IC9537	2	H1	H2	H1
IC9538	1	H2	H2	- ^d^
*Field C*				
Ascocarp 1				
IC10294	1	H1	H1	H1
IC10295	1	H1	H2	- ^d^
IC10296	1	H2	H2	- ^d^
Ascocarp 2				
IC10306	2	H2	H1	- ^d^
IC10307	1	H2	H1	H1
IC10308	2	H2	H2	H1

^a^NRRL strain numbers from the Agricultural Research Service Culture Collection, Peoria, Illinois, and IC strain numbers from the Ignazio Carbone Culture Collection, North Carolina State University, Raleigh, North Carolina.

^b^Due to the high number of triplets at the *AF48* locus in AF36, sequenced products range from 66 to 70 GAA repeats.

^c^PCR did not amplify.

^d^NRRL 21882 has a deletion of the entire aflatoxin gene cluster [11], which accounts for missing *aflC* in some of the progeny.

### Reciprocal crosses and mitochondrial inheritance

Reciprocal crosses between sclerotia and conidia were performed to elucidate female and male roles in sexual reproduction and to determine the pattern of mitochondrial inheritance. *A*. *flavus* sclerotia from single strains of one mating type formed ascospore-bearing ascocarps when incubated on the surface of sterilized soil inoculated with conidia of the opposite mating type, indicating that sclerotia functioned as female and conidia served as a male (Table 3). Hermaphroditism was shown by the formation of ascospores in sclerotia of both strains within each reciprocal cross. In the crosses NRRL 29507 × 21882 and NRRL 29473 × AF36, sclerotia from NRRL 29507 and NRRL 29473 (*MAT1-1*) readily formed ascospores when incubated with respective NRRL 21882 and AF36 (*MAT1-2*) conidia in the soil. However, fertility was significantly lower in reciprocal combinations in which NRRL 21882 and AF36 (*MAT1-2*) sclerotia were incubated with respective NRRL 29507 and NRRL 29473 (*MAT1-1*) conidia when compared by chi-square test for independence (P < 0.0001) (Table 3, S1 Text). In contrast, NRRL 29537 × 29536 showed markedly higher fertility when NRRL 29536 (*MAT1-2*) sclerotia were incubated with NRRL 29537 (*MAT1-1*) conidia compared to the reciprocal combination (P < 0.0001). None of the sclerotia-conidia combinations involving *MAT1-1* × *MAT1-1* and *MAT1-2* × *MAT1-2* produced ascocarps (Table 3).

The progeny from sclerotia-conidia crosses NRRL 29537 × NRRL 29536, NRRL 29507 × NRRL 21882 and NRRL 29473 × AF36 and their reciprocal combinations inherited the mitochondrial genome, as indicated by markers *AF-MIT-1*, *AF-MIT-3* and *AF-MIT-4*, respectively, from the sclerotial parent (Table 9). All sclerotia-conidia crosses exhibited segregation in the nuclear genome (*MAT* locus) of progeny (Table 9). Both mating-type alleles (*MAT1-1/MAT1-2*) were detected in eight of the progeny. Sequence data for mitochondrial *AF-MIT-1*, *AF-MIT-3* and *AF-MIT-4* were submitted to DRYAD database with accession DOI: http://dx.doi.org/10.5061/dryad.sk35h.

**Table 9:** Nuclear and mitochondrial loci for progeny from reciprocal crosses between single-strain sclerotia and conidia inoculated in sterile soil.

Strain^a^	Loci examined	
	Chr. 6 *MAT*	Mitochondria *AF-MIT-1*
**Parents**		
NRRL 29537 (sclerotia)	1	H1
NRRL 29536 (conidia)	2	H2
**Progeny**		
Ascocarp 1		
IC5813	1	H1
IC5814	2	H1
Ascocarp 2		
IC5825	1&2	H1
IC5826	2	H1
Ascocarp 3		
IC5837	2	H1
IC5838	1	H1
**Parents**		
NRRL 29537 (conidia)	1	H1
NRRL 29536 (sclerotia)	2	H2
**Progeny**		
Ascocarp 1		
IC5849	1	H2
IC5850	1&2	H2
Ascocarp 2		
IC5861	2	H2
IC5862	1&2	H2
Ascocarp 3		
IC5873	2	H2
IC5874	2	H2
Strain^a^	Chr. 6 *MAT*	Mitochondria *AF-MIT-3*
**Parents**		
NRRL 29507 (sclerotia)	1	H1
NRRL 21882 (conidia)	2	H2
**Progeny**		
Ascocarp 1		
IC5885	2	H1
IC5886	2	H1
Ascocarp 2		
IC5897	1&2	H1
IC5898	2	-^b^
Ascocarp 3		
IC5909	1&2	H1
IC5910	2	H1
**Parents**		
NRRL 29507(conidia)	1	H1
NRRL 21882 (sclerotia)	2	H2
**Progeny**		
Ascocarp 1		
IC5920	2	H2
IC5921	2	H2
Ascocarp 2		
IC5932	2	H2
IC5933	2	H2
Ascocarp 3		
IC5944	1	H2
IC5945	1&2	H2
Strain^a^	Chr. 6 *MAT*	Mitochondria *AF-MIT-4*
**Parents**		
NRRL 29473 (sclerotia)	1	H1
AF36 (conidia)	2	H2
**Progeny**		
Ascocarp 1		
IC5741	2	H1
IC5742	1	H1
Ascocarp 2		
IC5753	2	H1
IC5754	1	H1
Ascocarp 3		
IC5765	2	H1
IC5766	2	H1
**Parents**		
NRRL 29473 (conidia)	1	H1
AF36 (sclerotia)	2	H2
**Progeny**		
Ascocarp 1		
IC5777	1	H2
IC5778	1&2	H2
Ascocarp 2		
IC5789	1	H2
IC5790	1&2	H2
Ascocarp 3		
IC5801	1	H2
IC5802	2	H2

^a^NRRL strain numbers from the Agricultural Research Service Culture Collection, Peoria, Illinois, and IC strain numbers from the Ignazio Carbone Culture Collection, North Carolina State University, Raleigh, North Carolina.

^b^PCR did not amplify.

## Discussion

This research suggests that *A*. *flavus* is versatile in the manner in which sclerotia are fertilized and reproduce sexually on the soil surface. Single-strain sclerotia of one mating type, which appear to predominate in nature, can be fertilized by strains in native soil populations after dispersal from the crop. Furthermore, fertilized sclerotia without ascocarps, which originate from crops co-infected with sexually compatible strains, can also form the sexual stage after dispersal. Sexual reproduction in both single-strain and fertilized sclerotia was observed under laboratory conditions when incubated on soil containing natural fungal populations (Tables 1 and 4) and under field conditions on soil after one year (Table 2). Progeny from single-strain sclerotia exposed to natural soil populations in the laboratory and field showed the acquisition of novel alleles from outcrossing with soil strains as well as recombination (independent assortment of chromosomes) (Tables 5 and 7). In contrast, progeny from fertilized sclerotia applied to fields before ascocarp formation showed only known parental alleles (Table 8). Prior fertilization of sclerotia may have prevented additional fertilization events, as illustrated in basidiomycetes following dikaryon formation [35].

Outside of the present study, there are no known examples among Aspergilli in which a mature sclerotium is capable of being fertilized by natural soil populations. Additional research is needed to determine the nature of the soil propagule (conidium or hypha) and whether direct contact of the sclerotium with the propagule is required or chemotrophic growth of the propagule through soil to the sclerotium is involved. Receptor structures for fertilization have not been detected on the surface of *A*. *flavus* sclerotia. Heterothallic *Botrytis cinerea* commonly produces sclerotia that function as survival structures [36]. These sclerotia can also serve as a female parent but unlike *A*. *flavus*, *B*. *cinerea* produces specialized microconidia (spermatia) for fertilization [37]. Heterothallic *Epichloë* species produce conidia that act as spermatia for fertilizing immature stromata on grasses, but the conidia are transmitted primarily by insects and originate from other stromata rather than soil populations [38].

Laboratory incubation of single-strain sclerotia on soil resulted in wide variation in fertility despite an equal proportion of mating types in soil, with some strains readily producing ascospores and others showing little or no evidence of sexual reproduction (Table 1). This variation might be attributed to female fertility factors in the strains producing the sclerotia (see below), but also could be influenced by the degree of sexual compatibility between sclerotia and soil strains independent of mating type. Sexual reproduction in *Aspergillus* in general is regulated by over 70 genes at different stages of development [39]. Sexual incompatibility is typically due to an accumulation of mutations of these genes [40,41] and is expected to be most prevalent in asexual fungi that undergo extensive genetic drift [14,42,43]. *A*. *flavus* is predominantly asexual and populations comprise numerous clonal lineages of varying degrees of genetic relatedness [6,7,44]. Genetic incompatibilities among lineages in *A*. *flavus* may be partially responsible for the *MAT1-1* × *MAT1-2* crosses that exhibit low fertility or do not produce viable progeny [8,9]. In this study, the biocontrol strains NRRL 21882 and AF36 showed extremely low fertility when sclerotia were incubated on soil under laboratory and field conditions (Tables 1 and 2, respectively) but showed relatively high fertility when sclerotia were fertilized in specific crosses in culture slants (Table 4). Therefore, the low fertility with soil incubations may have been due to the genetic composition of the *A*. *flavus* populations, and exposure to different soil populations might result in higher fertility. A number of *A*. *flavus* progeny from laboratory and field experiments showed both *MAT1-1* and *MAT1-2* (Tables 5, 7 and 9). One such *MAT1-1/MAT1-2* progeny strain from a laboratory cross also was reported by Olarte et al. [9]. The mechanism responsible for the presence of both mating types is not understood and additional research is required to determine whether heterokaryosis, B chromosomes or ectopic plasmids are involved.

In both single-strain and fertilized sclerotia, incubation on soil with natural fungal populations showed much higher frequencies of ascospore formation under laboratory conditions of high relative humidity (100%) and a constant temperature (30°C) compared to suboptimal conditions in the fields. All three fields were exposed to similar temperatures (S1 Table) and contained *A*. *flavus* soil populations with both *MAT1-1* and *MAT1-2* strains. Furthermore, the three fields showed similar frequencies of ascospore formation in fertilized sclerotia from crosses (Table 2), suggesting that environmental conditions were similar and that differences in soil type (Faceville fine sandy loam in Field B and Greenville fine sandy loam in Fields A and C) had little effect. Despite the many similarities in field conditions, ascospore formation in single-strain sclerotia was observed at low frequencies only from Field B (Table 2). The most prominent difference among fields involved *A*. *flavus* L strain population densities in soil, with the density in Field B (8135 CFU/g) being approximately 20× higher than the densities in Fields A and C (Table 6). A high population density could increase the likelihood of sclerotium fertilization and account for the detection of sexual reproduction only in Field B.

Little is known about the mechanism of fertilization and the subsequent development of ascocarps, asci and ascospores in sclerotia of *A*. *flavus* and other section *Flavi* species. Gametangia have been reported in several *Aspergillus* species [45] but not in section *Flavi*. Homothallic *A*. *alliaceus* from section *Flavi* produces sclerotia whose matrix consists of thick-walled pseudoparenchymatous cells [46–48]. Fennell and Warcup [46] reported ‘channeling’ within the matrix immediately prior to the appearance of ascocarps. Transmission electron microscopy revealed that the channeling may be due to the interspersion of groups of cells containing cytoplasm with other groups of cells in various stages of autolysis [49]. Intracellular hyphae were also observed and may be involved in the early stages of ascocarp formation. In *A*. *flavus*, the matrix of mature sclerotia also consists of thick-walled pseudoparenchymatous cells with cytoplasmic contents [50]. Wada et al. [51] reported on the formation of heterokaryotic sclerotia in *A*. *oryzae*, a species often considered to be con-specific with *A*. *flavus* [52]. However, in that study, nutritional mutants were paired and sclerotia were produced independent of mating-type combination and only by strains with different auxotrophies.

In reciprocal crosses, sclerotia and conidia from both strains within each cross functioned as female and male, respectively, indicating *A*. *flavus* is hermaphroditic. The sclerotium, as a source of nutrients for sexual development, can be considered functionally female and the conidia used for fertilization functionally male. The degree of fertility depended upon the parental source of sclerotia and conidia (Table 3). In each reciprocal cross, one sclerotia-conidia combination was highly fertile while the reciprocal combination produced a significantly lower frequency (P < 0.0001) of sclerotia containing ascospore-bearing ascocarps. These results concur with the presence of fungal populations with varying proportions of strains that are hermaphroditic or female sterile [13,14,53], with three of the strains in this study (NRRL 29537, NRRL 21882, AF36) approaching female sterility (Table 3). The combinations leading to highest fertility involved sclerotia from both *MAT1-1* and *MAT1-2* strains, indicating that the gender-based sexual system is independent of the mating-type compatibility system [13]. Strain-dependent differential expression of sex-based genes necessary for fertilization and sexual development by female and male parents [54] could account for these differences and further work should be done to identify any causal link. Only three reciprocal sclerotia-conidia crosses of *A*. *flavus* were examined in this study and additional reciprocal crosses might reveal pairs of strains that show equal female fertility or strains in which one or both are completely female sterile.

Among the three pairs of reciprocal crosses between sclerotia and conidia, the sclerotial parent contributed the mitochondrial genome to progeny (Table 9). Such uniparental inheritance of mitochondria from the female parent is the most prevalent pattern in fungi [55]. Many anisogamous ascomycetes are characterized by an ascospore-bearing female whose nuclei are contributed to the ascocarp wall and whose nuclei and mitochondria are contributed to ascospores, and by a male whose nuclei are contributed solely to ascospores [14]. Outcrossing between strains in homothallic *A*. *nidulans* reveals such female and male roles in nuclear and mitochondrial inheritance during sexual reproduction [15] and is consistent with observations in *A*. *flavus*.

In conclusion, the current study helps to elucidate the mechanisms available to *A*. *flavus* for sexual reproduction in natural environments. Both proposed mechanisms—fertilization of single-strain sclerotia by native soil populations and fertilization of sclerotia in crops before dispersal onto soil—are supported by this research, though questions remain concerning their relative importance in nature. In addition, female fertility in *A*. *flavus*, as indicated through the reciprocal crosses, requires additional research to determine its role in regulating sexual reproduction and how it interacts with mating type in influencing population structure. Soil populations of *A*. *flavus* are highly diverse genetically [4,5,7]. Sweany et al. [56] reported that *A*. *flavus* populations in corn from 11 Louisiana fields comprised relatively few VCGs and haplotypes and were mostly *MAT1-*2, whereas soil populations from those same cornfields comprised a large number of VCGs and haplotypes and an equal proportion of mating types. Therefore, compared to *A*. *flavus* populations in crops, soil populations would provide a higher likelihood of exposure of sclerotia to sexually compatible strains and a more diverse source of genetic material for outcrossing.

## Supporting Information

S1 Table Weather conditions at three fields (2013–2014) where single-strain and fertilized sclerotia of *A*. *flavus* were applied. (DOCX)

S1 Text Statistics. (DOCX)
