# Supplementary material for: Sexual Reproduction in Aspergillus flavus Sclerotia: Acquisition of Novel Alleles from Soil Populations and Uniparental Mitochondrial Inheritance
Source: PLoS One. 2016 Jan 5;11(1):e0146169. doi: 10.1371/journal.pone.0146169 (PMC4701395; doi:10.1371/journal.pone.0146169)
Supplement: S1 Table — (DOCX) [file pone.0146169.s001.docx]

S1 Table. Weather conditions at three fields (2013-2014) where single-strain and fertilized sclerotia of *A. flavus* were applied.

_____________________________________________________________________________________________________________________

Air temperature (°C)^a^ Rainfall (cm)^b^

________________________________________________ ______________________________________________

Month Minimum Maximum Mean Field A Field B Field C

_____________________________________________________________________________________________________________________

2013

Apr 16-30 12.6 ± 0.2 25.9 ± 0.3 18.8 ± 0.1 3.8 (3) 3.6 (3) 6.7 (2)

May 15.2 ± 0.1 28.0 ± 0.6 21.5 ± 0.3 2.8 (5) 4.6 (5) 3.0 (4)

Jun 20.8 ± 0.3 31.5 ± 0.5 25.2 ± 0.2 25.4 (14) 15.5 (13) 16.2 (15)

Jul 21.1 ± 0.5 30.8 ± 0.5 24.6 ± 0.3 20.9 (21) 20.2 (21) 26.2 (18)

Aug 20.8 ± 0.6 31.5 ± 0.5 25.2 ± 0.3 15.7 (8) 17.6 (8) 20.0 (8)

Sep 19.0 ± 0.4 30.0 ± 0.4 23.6 ± 0.3 5.8 (7) 5.5 (5) 7.1 (8)

Oct 13.4 ± 0.5 26.1 ± 0.4 19.1 ± 0.3 3.9 (2) 3.0 (2) 7.8 (1)

Nov 7.1 ± 0.3 19.0 ± 0.2 12.5 ± 0.2 7.1 (4) 9.9 (4) 7.2 (3)

Dec 6.3 ± 0.1 17.6 ± 0.2 11.5 ± 0.1 19.5 (10) 18.6 (10) 19.6 (9)

2014

Jan -0.8 ± 0.3 11.8 ± 0.1 5.1 ± 0.2 7.0 (11) 8.1 (12) 10.7 (11)

Feb 4.9 ± 0.3 17.1 ± 0.2 10.8 ± 0.1 11.9 (9) 13.4 (9) 14.8 (9)

Mar 6.1 ± 0.1 19.4 ± 0.3 12.7 ± 0.1 10.0 (8) 11.9 (8) 12.5 (8)

Apr 1-15 11.3 ± 0.4 24.5 ± 0.4 17.8 ± 0.2 17.6 (9) 14.1 (7) 15.2 (4)

_____________________________________________________________________________________________________________________

^a^Means ± SD (n = 3) based on monthly means of three fields; temperatures for each field were calculated from daily temperatures for each month (n = 28-31 days), except for April (n = 15).

^b^Numbers in parentheses are number of days per month in which rainfall exceeded 1 mm.
