# Supplementary material for: Sexual Reproduction in Aspergillus flavus Sclerotia: Acquisition of Novel Alleles from Soil Populations and Uniparental Mitochondrial Inheritance
Source: PLoS One. 2016 Jan 5;11(1):e0146169. doi: 10.1371/journal.pone.0146169 (PMC4701395; doi:10.1371/journal.pone.0146169)
Supplement: S1 Text — (DOCX) [file pone.0146169.s002.docx]

**S1 Text. Statistics.**

**Table 1**

Total number of fertile sclerotia and unfertile sclerotia for each sclerotium-producing strain was calculated by summing the replicates. Chi-square test of independence was run using Microsoft Excel 2013 CHISQ.TEST function for pairwise comparisons of fertility between sclerotium-producing strains. Comparisons with *P* < 0.05 were considered to show significant differences in fertility.

|  | NRRL 29507 | NRRL 29473 | NRRL 29537 | NRRL 29536 | NRRL 21882 | NRRL 29487 | AF36 |
| --- | --- | --- | --- | --- | --- | --- | --- |
| NRRL 29507 | - | 0.51 | 9.51E-112 | 4.47E-09 | 7.50E-106 | 7.78E-107 | 5.74E-107 |
| NRRL 29473 | - | - | 1.02E-105 | 7.66E-11 | 5.22E-100 | 4.51E-101 | 3.37E-101 |
| NRRL 29537 | - | - | - | 4.33E-167 | 0.30 | 0.33 | 0.33 |
| NRRL 29536 | - | - | - | - | 9.66E-160 | 5.00E-160 | 3.31E-160 |
| NRRL 21882 | - | - | - | - | - | 0.09 | 0.09 |
| NRRL 29487 | - | - | - | - | - | - | 1.00* |
| AF36 | - | - | - | - | - | - | - |

*Comparison between AF36 and NRRL 29487 performed with rxc XML on Mobyle SNAP Workbench [34].

**Table 2**

We tested using a hypergeometric test whether we would expect to see the number of fertile sclerotia in Field B if the three fields (A, B and C) were equally likely to produce fertile sclerotia. The hypergeometric distribution models the probability of *i* successes in a sample of *k* given a population of size *N* with *n* successes and *m* failures. If all three fields are identical, this model represents the probability of a field (*k*) producing a number of fertile single-strain sclerotia (*i*). Each application of single-strain sclerotia represents an individual in the population (*N* = 21). Applications that produced any fertilized sclerotia are successes (*n* = 3) while applications in which fertile sclerotia were not found are failures (*m* = 18). The probability of one field (*k* = 7) containing all 3 fertile sclerotia applications (*i* = 3) is *P* = 0.03, calculated using Microsoft Excel 2013 HYPGEOM.DIST function.

**Table 3**

Total number of fertile sclerotia and unfertile sclerotia for each sclerotial strain × conidial strain cross was calculated by summing the replicates. Chi-square test of independence was run using Microsoft Excel 2013 CHISQ.TEST function for pairwise comparisons of fertility within reciprocal crosses. Comparisons with *P* < 0.05 were considered to show significant differences in fertility.

| *MAT1-1* sclerotial strain x *MAT1-2* conidial strain | *MAT1-2* sclerotial strain x *MAT1-1* conidial strain | *P*-value |
| --- | --- | --- |
| NRRL 29537 × NRRL 29536 | NRRL 29536 × NRRL 29537 | 1.34E-162 |
| NRRL 29473 × AF36 | AF36 × NRRL 29473 | 3.81E-24 |
| NRRL 29507 × NRRL 21882 | NRRL 21882 × NRRL 29507 | 1.07E-61 |

**Table 4**

Total number of fertile sclerotia and unfertile sclerotia for each cross was calculated by summing the replicates. Chi-square test of independence was run using Microsoft Excel 2013 CHISQ.TEST function for pairwise comparisons of fertility between crosses in which sclerotia were incubated for 4 mo in culture slants and in nonsterile soil cups. Comparisons with *P* < 0.05 were considered to show significant differences in fertility.

| Culture slants (4 mo) |  |  |  |  |  |  |
| --- | --- | --- | --- | --- | --- | --- |
|  | NRRL 29473 × NRRL 29487 | NRRL 29537 × NRRL 29536 | NRRL 29507 × AF36 | NRRL 29473 × AF36 | NRRL 29507 × NRRL 21882 | NRRL 29473 × NRRL 21882 |
| NRRL 29473 × NRRL 29487 | - | 4.99E-40 | 2.03E-144 | 6.83E-99 | 1.12E-47 | 9.73E-06 |
| NRRL 29537 × NRRL 29536 | - | - | 2.01E-50 | 2.13E-19 | 0.17 | 4.53E-20 |
| NRRL 29507 × AF36 | - | - | - | 7.27E-13 | 2.76E-43 | 8.81E-111 |
| NRRL 29473 × AF36 | - | - | - | - | 1.58E-14 | 1.43E-68 |
| NRRL 29507 × NRRL 21882 | - | - | - | - | - | 8.32E-26 |
| NRRL 29473 × NRRL 21882 | - | - | - | - | - | - |

| Nonsterile soil cups (4 mo) |  |  |  |  |  |  |
| --- | --- | --- | --- | --- | --- | --- |
|  | NRRL 29473 × NRRL 29487 | NRRL 29537 × NRRL 29536 | NRRL 29507 × AF36 | NRRL 29473 × AF36 | NRRL 29507 × NRRL 21882 | NRRL 29473 × NRRL 21882 |
| NRRL 29473 × NRRL 29487 | - | 0.0004 | 9.54E-19 | 2.90E-10 | 9.14E-16 | 1.69E-05 |
| NRRL 29537 × NRRL 29536 | - | - | 3.88E-08 | 0.005 | 1.68E-06 | 1.02E-14 |
| NRRL 29507 × AF36 | - | - | - | 0.006 | 0.38 | 1.09E-36 |
| NRRL 29473 × AF36 | - | - | - | - | 0.03 | 5.89E-25 |
| NRRL 29507 × NRRL 21882 | - | - | - | - | - | 1.95E-29 |
| NRRL 29473 × NRRL 21882 | - | - | - | - | - | - |
